# A Whole-Genome Sequencing-Based Study to Delineate the Risk and Characteristics of Tuberculosis Transmission in an Insular Population Over 10 Years in Shanghai

**DOI:** 10.3389/fmicb.2021.768659

**Published:** 2022-02-16

**Authors:** Min Wang, Yangyi Zhang, Cheng Huang, Jing Li, Xin Shen, Genming Zhao, Yuan Jiang, Qichao Pan

**Affiliations:** ^1^Division of TB and HIV/AIDS Prevention, Shanghai Municipal Center for Disease Control and Prevention, Shanghai, China; ^2^Department of Epidemiology, School of Public Health and Key Laboratory of Public Health Safety, Fudan University, Shanghai, China; ^3^Department of Tuberculosis Control, Chongming District Center for Disease Control and Prevention, Shanghai, China

**Keywords:** tuberculosis, transmission, whole-genome sequencing, diagnostic delay, genomic cluster, epidemiological investigation

## Abstract

**Background:**

Tuberculosis (TB) has remained a tough problem in China. This study aims to identify the risk of tuberculosis transmission and to assess its characteristics.

**Methods:**

We performed a molecular epidemiological study for patients with culture-positive *Mycobacterium tuberculosis* (*M. tuberculosis*) in Shanghai, from 2009 to 2018. Demographic information was obtained from the Tuberculosis Information Management System. Whole-genome sequencing (WGS) was conducted with a threshold of 12 single-nucleotide polymorphisms (SNPs) to distinguish the genomic cluster. To analyze the characteristics of TB transmission, the contact investigation for clustered cases was performed.

**Results:**

In total, 94 (27.25%) of the 345 enrolled patients were grouped into 42 genomic clusters, indicating local transmission of *M. tuberculosis* strains. Compared to a health system delay <14 days, patients with a health system delay ≥14 days [adjusted odds ratios (AOR) = 2.57, 95% confidence interval (CI): 1.34–4.95] were more likely to be clustered. Patients under 65 years old (AOR = 3.11, 95% CI: 1.76–5.49), residents (AOR = 2.43, 95% CI: 1.18–4.99), and Beijing genotype strains (AOR = 3.35, 95% CI: 1.32–8.53) were associated with increased risk of clustering. Interestingly, patients with resistance to isoniazid (AOR = 2.36, 95% CI: 1.15–4.88) had a higher risk of transmission. Sixteen confirmed/probable epidemiological links were identified. Local transmission of imported cases and household transmission were prominent.

**Conclusion:**

Health system delay is a crucial factor for TB transmission. Patients with resistance to isoniazid should be priority targets for contact investigation to reduce transmission.

## Introduction

Tuberculosis (TB) is a chronic communicable disease and remains a serious public threat worldwide. Despite great efforts at curbing the spreading of *Mycobacterium tuberculosis* (*M. tuberculosis*), the WHO has estimated that, of the 10.0 million (range, 8.9–11.0 million) people who fell ill with TB, an estimated 1.2 million TB-related deaths were among HIV-negative people in 2019 (World Health Organization [WHO], 2020).

Standard TB infection control focuses on patients already on treatment, but the spread of TB should not be ignored under frequent social activities. Transmission leads to new infections and cases. Next-generation sequencing technologies have facilitated the development of molecular epidemiology, and we can now study the transmission of TB at a high resolution (Gagneux, 2017). Although molecular approaches can help differentiate between reactivation or reinfection among TB cases, whole-genome sequencing (WGS) has been proven invaluable to illustrate the network and direction of transmission in recent years (Walker et al., 2014; Comin et al., 2020).

Identifying genomic clusters and understanding the transmission dynamics may be helpful to interpret the networks of TB spreading (Anderson et al., 2014; Walker et al., 2014; Kruczak et al., 2019). However, most previous WGS-based studies have only focused on exploring the transmission of multidrug-resistant/drug-resistant tuberculosis (MDR/DR-TB) (Clark et al., 2013; Senghore et al., 2017; Yang et al., 2017; Bouzouita et al., 2019; Dixit et al., 2019; Jiang et al., 2019) or describing the dynamics of TB in metropolitan cities under mass migrations (Yang et al., 2018; Abascal et al., 2019; Jiang et al., 2019). To develop tailored control strategies, it is essential to evaluate the real situation of TB transmission in a specific context (Auld et al., 2017). Few studies fully delineate the TB transmission in a relatively stable population and exclude the impact of population migration.

Factors that contribute to TB transmission could increase the risk of the population acquiring TB, which remains a threat to end TB. Identifying the risk factors and taking target measures may be helpful to reduce the morbidity of TB. Also, despite the advance in knowing the importance of early case finding and treatment, millions of TB cases are being missed due to health system delay, which results in sustained transmission (Floyd et al., 2018). Health system delay is the time interval between patients’ first seeking medical care and the date of diagnosis (Getnet et al., 2017), which has been reported to be longer in smear-negative or extrapulmonary TB (Broderick et al., 2018; Datiko et al., 2020). However, the role of the health system delay contributing to the dissemination of the disease is not known yet.

Chongming is a suburban island in Shanghai with a unique geographical location and limited population movement. About 0.678 million people live in 18 townships, and Shanghai is promoting this district as a world-class ecological island. Although several control measures have been implemented, the morbidity of TB declined slowly. To delineate the risk and characteristics of TB transmission in a relatively insular population, we conducted a WGS-based epidemiological study for all *M. tuberculosis* isolates in this district over a 10-year period.

## Materials and Methods

### Sample Source and Study Population

In China, all suspected pulmonary TB patients are referred to local TB-designated hospitals for diagnosis, treatment, and registration management. Through a routine surveillance system, the Shanghai Municipal Centre for Disease Control and Prevention (CDC) collected and preserved all culture-positive *M. tuberculosis* isolates from local TB-designated hospitals. However, the isolates of patients who were first diagnosed in the Shanghai Pulmonary Hospital were not sent to the Shanghai CDC. Our study obtained all clinical isolates of TB patients in the Chongming district, which were collected from the Shanghai CDC.

This study included patients aged 15 years or older with culture-confirmed pulmonary TB reported in Shanghai, between January 1, 2009 and December 31, 2018. Demographic information of TB patients was collected from the Tuberculosis Information Management System, which has updated small modules according to the work needs during the 10 years. Despite this, the main data collected in our study were not affected, such as age, sex, residential address, diagnostic time, occupation, and census register (residents refer to local patients with census registration in the Chongming district rather than migrants from other provinces in China).

Phenotypic drug susceptibility testing (DST) against first-line anti-tuberculosis drugs was performed using the proportion method on Lowenstein–Jensen medium at the following concentrations: rifampicin 40.0 μg/ml, isoniazid 0.2 μg/ml, streptomycin 4.0 μg/ml, and ethambutol 2.0 μg/ml, respectively (World Health Organization [WHO], 2018).

### Definitions

#### Genomic Cluster

*Mycobacterium tuberculosis* isolates with a genomic difference (s) ≤ 12 single-nucleotide polymorphisms (SNPs) were defined as a genomic cluster (Yang et al., 2017).

#### Health System Delay

The time interval between patients’ first consultation with TB-designated hospitals and the date of diagnosis (Getnet et al., 2017).

#### A Bacteriologically Confirmed Tuberculosis Case

One from whom a biological specimen is positive by smear microscopy, culture, or WHO-approved rapid diagnostics (such as Xpert MTB/RIF).

#### Tuberculosis-Designated Hospital

Refers to the medical institution designated by the administrative department of health to engage in the diagnosis, treatment, report registration, follow-up inspection, and management of tuberculosis during treatment in China (Beijing Municipal Health Commission [BMHC], 2021).

#### Confirmed Epidemiological Links

Patients knew each other and have confirmed relationships such as family, workmates, schoolmates, friends, and so on (Yang et al., 2017).

#### Probable Epidemiological Links

Patients did not know each other but shared locations where TB transmission probably occurred, including in a neighborhood complex or lived in the same town (Yang et al., 2017).

#### No Epidemiological Links

Patients did know each other, did not live in the same town, or lacked a common neighborhood or setting.

### Whole-Genome Sequencing and Bioinformatics Analysis

Extraction and purification of genomic DNA were carried out following QIAamp DNA Mini Kit (Qiagen) protocols. Libraries were constructed by Nextera XT library prep (Illumina), and paired-end 150-bp DNA sequencing was performed on a Hiseq 2500 platform (Illumina) with an expected coverage of 100×. The bioinformatics analysis was performed following a previously validated pipeline (Yang et al., 2018). *M. tuberculosis* H37Rv strain (GenBank accession number NC_000962.3) was used as the reference template. Sequencing reads were mapped to the reference genome using Bowtie2 (v2.3.1). SAMtools (v1.6) was used for SNP calling with mapping quality greater than 30. Fixed mutations (frequency ≥ 75%) were identified using VarScan (v2.3.6) with at least 10 reads supporting and the strand bias filter option on. We excluded all SNPs annotated in regions, such as PPE/PE-PGRS family genes, phage sequences, insertion, or mobile genetic elements (Casali et al., 2016).

To assess the genetic drug resistance, the data of SNPs were compared with a list of the mutations related to anti-TB drug resistance (Coll et al., 2015). Excluding mutations in drug-resistance-associated genes, all SNP locations were combined into a non-redundant consensus list and were recalled with the mpileup2cns function of VarScan to build a phylogenetic tree. Nucleotide positions with missing calls present on more than 5% of the isolates were removed (Liu et al., 2018; Liu et al., 2020). A phylogenetic tree was constructed with MEGA X (version 10.1) using the maximum-likelihood method and a general time-reversible model of nucleotide substitution, with a gamma distribution (GTRGAMMA) and 500 bootstraps. Visualization of the bacteriological information was performed at Interactive Tree of Life.^1^ We adapted a recently described hierarchical nomenclature to define nodes and subclades within the tree in the definition of sub-lineages (Coll et al., 2014).

### Epidemiological Investigation

Clustered cases were invited to participate in a contact investigation. After acquiring the informed consent, we have conducted a questionnaire in person or telephone interviews. To understand the social relationship between clustered cases, we collected data on close contacts, social contacts, and places they frequented before their diagnosis of TB, including the information of medical records, workplace, and residential address.

### Statistical Analysis

We compared the characteristics between “clustered” and “non-clustered” using the chi-square test. Multivariate logistic regression was conducted to identify the risk factors of TB transmission, such as demographic, bacteriological, phenotypic resistance, and clinical characteristics. Factors with a *P*-value less than 0.05 in the final model were independently associated with genomic clusters. The adjusted odds ratios (AORs) and 95% confidence intervals (95% CIs) were calculated. Our statistical analysis was conducted with SAS (version 9.4). The clustering rate was calculated as the percentage of clustered patients among the number of total patients (number of clustered patients/number of total patients).

### Ethics Approval and Institutional Safety Procedures

This study protocol was approved by the Ethical Review Committee at the Shanghai CDC (No. 2020-14). All participants provided written informed consent after completing the description of the study.

All biological experimentations in our study were performed after acquiring approval from the Biosafety Commission, Shanghai CDC. Our study abides by *National Rules on Biosafety Management of Pathogenic Microorganism Laboratory* (State Council of the People’s Republic of China [SCPRC], 2018) to conduct all specific procedures, such as microscopy, culture, DST, and molecular testing. A set of essential biosafety measures was enacted to minimize risks in our TB laboratory, including risk assessment, plans for emergency preparedness and response, codes of practice, personal protective equipment and clothing, as well as disposal procedures for contaminated materials.

### Genomic Data Availability

Sequence data associated with this study were deposited in the Sequence Read Archive (SRA) of NCBI under project accession PRJNA760838.

## Results

### Basic Information

**FIGURE 1:**
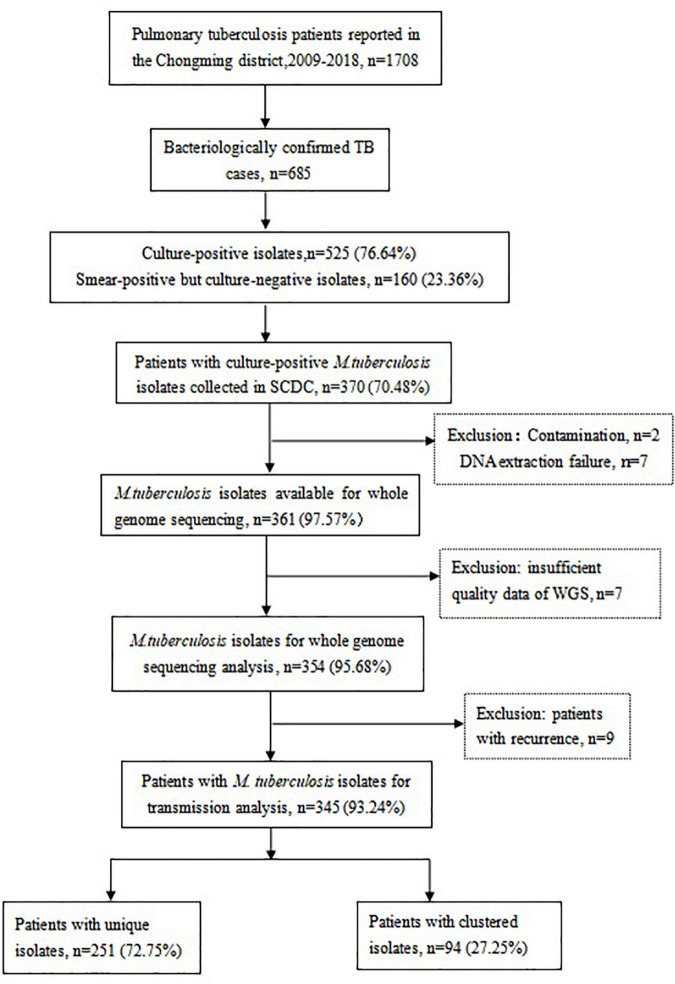
Flow chart of study population and selection.

As shown in Figure 1, 1,708 TB cases were reported in the Chongming district between 2009 and 2018, including 685 bacteriologically confirmed TB cases. There were 525 culture-positive isolates and 160 smear-positive but culture-negative isolates. Of those, 370 patients (70.48%, 370/525) with culture-positive *M. tuberculosis* isolates were enrolled in this study. The rest of patients (155 culture-positive isolates) who were first diagnosed in the Shanghai Pulmonary Hospital were not included. In total, 16 isolates were excluded due to DNA extraction failure, contamination, or insufficient quality of WGS data. Seven patients had a relapse of pulmonary TB and one patient relapsed twice, so we excluded the nine isolates of relapsed patients. For the final analysis, 345 *M. tuberculosis* isolates were included.

The median age of the TB patients was 60 years (range 38–74). A total of 277 patients (80.29%, 277/345) were residents with census register in the Chongming district. About 86.09% of the patients (297/345) were new TB cases. The percentage of patients with resistance to isoniazid (INH) was 12.46% (43/345). The median time of health system delay was 3 days (range 2–9 days).

**FIGURE 2:**
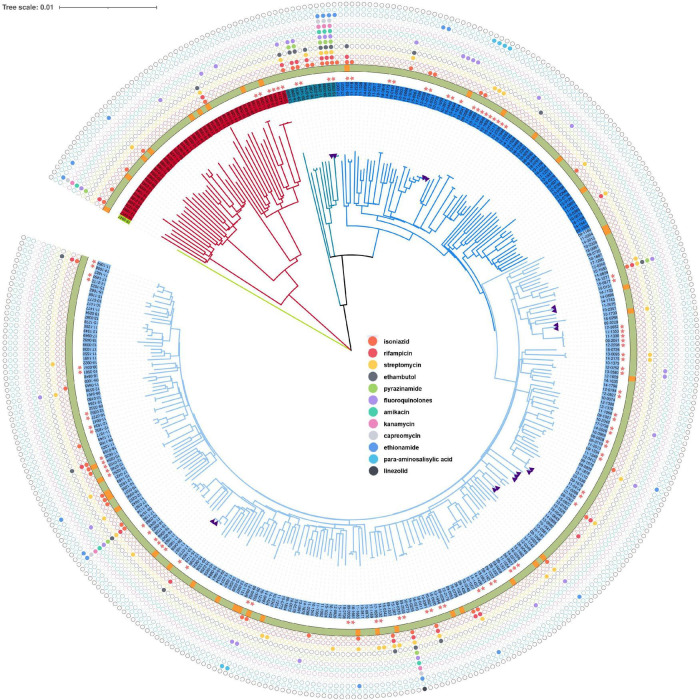
Phylogenetic tree for the 354 *Mycobacterium tuberculosis* isolates from Shanghai, China, 2009–2018. The purple triangles represent isolates from relapsed cases (*n* = 9). The pink five-pointed stars indicate the clustered isolates, whose genetic distance is ≤12 single-nucleotide polymorphisms (SNPs). The outer ring shows the census register of patients, of which the green represents resident and the orange represents migrant. From inside to outside, the solid colorful circles represent drug resistance mutations for isoniazid, rifampicin, streptomycin, pyrazinamide, amikacin, kanamycin, capreomycin, ethionamide, *para*-aminosalicylic acid, and linezolid.

Most of the *M. tuberculosis* isolates belonged to the lineage 2 (*n* = 298, 86.38%) followed by lineage 4 (*n* = 46, 13.33%), and lineage 3 (*n* = 1, 0.29%). Sub-lineage 2.2.1 (*n* = 289, 83.76%) was the main sub-lineage. The phylogenetic tree revealed that three lineages of *M. tuberculosis* isolate circulated in the Chongming district (Figure 2). The tree also displays the drug resistance profile for 12 anti-TB drugs based on the presence of validated resistance-conferring mutations. The mutations in drug resistance genes mainly occurred in *rpsL*, *katG*, and *rpoB*.

### The Clustering Rate

Among 345 TB cases, 94 were grouped into 42 genomic clusters. The clustering rate was 27.25%, which indicated TB transmission in this district. The genomic clusters consisted of two to five strains, and the majority of the clusters had two strains, accounting for 74.47% (Table 1) of the clustered cases (70/94).

**TABLE 1:** The cluster size and the number of genomic clusters of 94 *Mycobacterium tuberculosis* isolates.

No of isolates in clusters (N)	No of clusters (N)	No of isolates (N)	Proportion (%)
2	35	70	74.47
3	5	15	15.96
4	1	4	4.26
5	1	5	5.32
Total	42	94	100.00

### Risk Factors Associated With Genomic Clusters

Beijing genotype strains were more frequent in clustered cases than in non-clustered cases (*P* < 0.05). Resistance to isoniazid was associated with genomic clusters (*P* < 0.05). Additionally, positive sputum smears and cavitation on chest radiography did not differ between clustered cases and non-clustered cases (*P* > 0.05) (Table 2).

**TABLE 2:** Demographic, bacteriological, phenotypic resistance, and clinical characteristics of genomic clustered and non-clustered cases in Shanghai, China, 2009–2018.

Characteristics	Clustered (*N* = 94)		Non-clustered (*N* = 251)		Total (*N* = 345)		χ2	*P*
	*N*	%	*N*	%	*N*	%		
**Sex**							0.36	0.55
Male	79	84.04	204	81.27	283	82.03		
Female	15	15.96	47	18.73	62	17.97		
Age* (y)								
15–24	15	15.96	27	10.76	42	12.17	Ref	–
25–34	10	10.64	24	9.56	34	9.85	0.34	0.56
35–44	7	7.45	17	6.77	24	6.96	0.29	0.59
45–54	14	14.89	28	11.16	42	12.18	0.05	0.82
55–64	23	24.47	31	12.35	54	15.65	0.47	0.49
65≥	25	26.60	124	49.40	149	43.19	6.77	0.01
**Census register**							3.94	**0.04**
Resident	82	87.23	195	77.69	277	80.29		
Migrant	12	12.77	56	22.31	68	19.71		
**Occupation**							4.50	**0.03**
Student	11	11.70	13	5.18	24	6.96		
Non-student	83	88.30	238	94.82	321	93.04		
**TB-history**							1.88	0.17
Yes	17	18.09	31	12.35	48	13.91		
No	77	81.91	220	87.65	297	86.09		
**Beijing genotype strains**							5.76	**0.02**
Yes	88	93.62	210	83.67	298	86.38		
No	6	6.38	41	16.33	47	13.62		
**MDR-TB^†^**								0.26
Yes	7	7.45	10	3.98	17	4.93		
No	87	92.55	241	96.02	328	95.07		
**Resistance to INH**							7.11	**0.01**
Yes	19	20.21	24	9.56	43	12.46		
No	75	79.79	227	90.44	302	87.54		
**Drug resistance**							6.43	**0.01**
Yes	22	23.40	31	12.35	53	15.36		
No	72	76.60	220	87.65	292	84.64		
**Health system delay**							6.45	**0.01**
<14 days	71	75.53	218	86.85	289	83.77		
≥14 days	23	24.47	33	13.15	56	16.23		
Source of patients**							1.95	0.38
Clinical consultation	35	37.23	98	39.8	133	39.00		
Referral	58	61.70	140	56.68	198	58.06		
Others	1	1.06	9	3.64	10	2.93		
Complications**							0.42	0.51
Yes	7	7.45	24	9.72	31	9.09		
No	87	92.55	223	90.28	310	90.91		
Sputum smear**							0.56	0.45
Yes	63	67.74	174	71.9	237	70.75		
No	30	32.26	68	28.1	98	29.25		
Cavity**							0	0.99
Yes	33	35.11	85	35.12	118	35.12		
No	61	64.89	157	64.88	218	64.88		

*Univariate logistic analysis, Ref, reference.

*^†^Represents the P-value calculated by Fisher’s exact probability method.*

**The clinical information of some samples was missing.

*The bold values mean these factors were statistically significant.*

In the multivariable logistic analysis, factors independently associated with genomic cluster included age, census register, health system delay, genotype, and resistance to isoniazid. As shown in Table 3, patients under 65 years old were more likely to be clustered than patients aged 65 years or older (AOR = 3.11, 95% CI: 1.76–5.49). Residents (AOR = 2.43, 95% CI: 1.18–4.99) and Beijing genotype strains (AOR = 3.35, 95% CI: 1.32–8.53) had a higher risk of clustering. Interestingly, patients with resistance to isoniazid were more likely to be clustered than others (AOR = 2.36, 95% CI: 1.15–4.88). Compared to a health system delay < 14 days, a health system delay ≥ 14 days increases by 2.57 (95% CI: 1.34–4.95) times the risk to form genomic clusters.

**TABLE 3:** Univariate and multivariable regression analysis on the risk factors of genomic clustering.

Characteristics	Total (*N*)	Clustered (*N*/%)	Univariate analysis		Multivariable regression	
			COR (95% CI)	*P*	AOR (95% CI)	*P*
**Age** (y)						
<65	196	69 (35.20)	2.69 (1.60, 4.53)	**<0.01**	3.11 (1.76, 5.49)	**<0.0001**
≥65	149	25 (16.78)	Ref			
**Occupation**						
Student	24	11 (45.83)	2.43 (1.05, 5.63)	**0.03**	1.54 (0.62, 3.84)	0.35
Non-student	321	83 (25.86)	Ref			
**Census register**						
Resident	277	82 (29.60)	1.96 (0.99, 3.85)	**0.04**	2.43 (1.18, 4.99)	**0.02**
Migrant	68	12 (17.65)	Ref			
**Health system delay**						
≥14 days	56	23 (41.07)	2.14 (1.18, 3.88)	**0.01**	2.57 (1.34, 4.95)	**0.01**
<14 days	289	71 (24.57)	Ref			
**Beijing genotype strains**						
Yes	298	88 (29.53)	2.86 (1.17, 6.99)	**0.02**	3.35 (1.32, 8.53)	**0.01**
No	47	6 (12.77)	Ref			
**Resistance to INH**						
Yes	43	19 (44.19)	2.40 (1.24, 4.62)	**0.01**	2.36 (1.15, 4.88)	**0.02**
No	302	75 (24.83)	Ref			

*The bold values mean these factors were statistically significant.*

### Epidemiological Investigation and Pathway of Tuberculosis Transmission

Thirty-three clustered cases were investigated, with a response rate of 35.11% (33/94), and those cases covered 52.38% of the genomic clusters (22/42). We found four clusters with confirmed epidemiological links, in which one cluster included two prisoners from the same prison and three clusters included six family members. Based on the residential address, we found that several patients in the same clusters lived in the same towns. Thus, probable epidemiological links could be established for 28 patients from 12 clusters. No epidemiological links were found in eight genomic clusters.

Many clustered patients reported a history of exposure to TB with family members (C11, C12, and C25) or classmates (C02, C15, C16, and C26). There were five patients of four clusters (C02, C15, C24, and C29) that worked in other places before they were diagnosed. The WGS data indicated that they were in the same clusters with other residents. Several clustered cases were from the same sex, shared the same occupation, and had similar age. The young people often went to internet cafes and school, while the older group often went to markets, chess, and card rooms. It means that casual contact transmission could occur in those same groups due to their lifestyles.

## Discussion

### Summary of the Principal Findings

This genomic epidemiological study systematically delineates the relative contribution of TB transmission and identifies principal risk factors that may be responsible for TB transmission in Shanghai, from 2009 to 2018. Our study concludes that health system delay and resistance to isoniazid are principal contributing factors for the dissemination of the disease. A total of 27.25% (94/345) clustered cases were identified, implying that local transmission is a threat to end TB. WGS was performed for all available *M. tuberculosis* isolates in a 10-year period in the Chongming district, Shanghai, which could accurately reflect the level of TB transmission.

Our study shows the capability of WGS to identify exact clusters. The epidemiological investigation has further validated that confirmed or probable epidemiological links exist in some genomic clusters. We have found 16 clusters with confirmed/probable epidemiological links by contact investigation. Based on the medical records, WGS data, and epidemiological information, the transmission relationships of clustered patients were explored, and the characteristics of TB transmission were summarized. Household transmission is an important characteristic in this area. Several patients in the same cluster share family relationships, and many clustered patients have a history of family contact with TB.

### Significance of the Study

Our study suggested that patients under 65 years old or residents had a higher risk for transmission. To halt the spread of TB, active case finding should be focused on these high-risk groups. A study in the United Kingdom suggests that most patients who migrate from high-incidence countries do not lead to local transmission (Walker et al., 2014). However, our results are distinct from a study in the Songjiang district, Shanghai, which inferred that TB transmission from residents to migrants occurs as commonly as transmission from migrants to residents (Yang et al., 2018). One possible explanation is that older residents in the Chongming district have lower awareness of TB and have more frequent social contact with others, which increases the chance to be infected and transmit to others. Also, most migrants in the Songjiang district could be younger groups with stronger bodies.

This study noted that Beijing genotype strains had a higher risk of clustering. This finding is consistent with previous studies (Yang et al., 2015; Jiang et al., 2019), indicating that the Beijing genotype may have increased transmissibility. However, it contrasts with a study (Lalor et al., 2017) conducted in England, which noted that the Beijing genotype strains are not associated with transmissibility among household contacts.

We detected a significant association between resistance to isoniazid and genomic clusters. A previous study (Dheda et al., 2017) has showed that close contacts of drug-resistant TB cases are more likely to be infected with a more infectious *M. tuberculosis* strain. A recent study (Theron et al., 2020) reported similar results, showing that drug-resistant TB patients have higher numbers of culturable *M. tuberculosis* in an aerosol. Our results also showed that 20.21% (19/94) of clustered isolates were resistant to isoniazid. A systematic review reported that treatment of isoniazid-resistant TB with the standardized first-line regimen led to treatment failure in 11% of the cases, compared with 2% among drug-susceptible TB patients (Gegia et al., 2017).

In our study, patients with a health system delay ≥ 14 days have a higher risk to transmit the disease. Chongming district is at the mouth of the Yangtze River, with relatively inconvenient traffic and a low economic level. This delay may be due to the unavailability of rapid and accurate diagnostic technologies required to confirm TB. A study (Segagni Lusignani et al., 2013) indicated that living in rural areas is associated with prolonged health system delay. It is worth noting that the diagnostic delay could prolong the infectious period. A meta-analysis (Bao et al., 2019) revealed that the case finding delay is a major contributing factor to the spread of TB in outbreaks. Also, the diagnostic delay might result in worse treatment outcome and increased risk of transmission (Cheng et al., 2013; Shiferaw and Zegeye, 2019). However, the main diagnostic tests for TB are rapid molecular assays, smear microscopy, and culture-based methods. Culture-based methods are the reference standard, which require a higher level of TB laboratory and can take up to 8 weeks to get results (World Health Organization [WHO], 2020). To achieve early diagnosis and promote early treatment, health system delay should be reduced by introducing rapid and accurate diagnostic tools such as sequencing technologies.

### Strengths and Limitations of the Study

Our study has several limitations and strengths. First, the level of TB transmission may be underestimated. About 30% (155/525) of culture-positive isolates were not collected in our study. Also, it is unable to analyze the TB transmission of patients without clinical strains. Second, we obtained limited information about clustered cases due to the retrospective study design. Therefore, epidemiological links between clustered cases were difficult to interpret clearly. However, this was the first population-based study using WGS to explore the transmission of TB in the Chongming district, Shanghai. Also, the long-span study timeframe facilitates the capture of all TB cases and provides insights into TB transmission dynamics. We have traced the infection source of the population studied at the molecular level, and the transmission chains of TB patients have been detected.

### Implications for Practice or Policy

Health system delay is a crucial factor for the transmission of TB in rural areas. The public health strategies should be redirected to reduce health system delay by introducing rapid and accurate diagnostic tools. Additionally, it could be feasible to take targeted interventions on patients with resistance to isoniazid.

## Data Availability Statement

The original contributions presented in the study are publicly available. This data can be found here: https://www. ncbi.nlm.nih.gov/bioproject/?term=PRJNA760838.

## Ethics Statement

The studies involving human participants were reviewed and approved by Ethical Review Committee of Shanghai Municipal Center for Disease Control and Prevention. Written informed consent to participate in this study was provided by the participants’ legal guardian/next of kin.

## Author Contributions

MW, YZ, and CH performed the material preparation, data collection, and data analysis. MW wrote the first draft of the manuscript. YZ, JL, and XS helped to proofread the manuscript. QP, GZ, and YJ supervised the study and made a critical revision to the manuscript. All authors contributed to the study conception and design, and approved the submitted version.

## Conflict of Interest

The authors declare that the research was conducted in the absence of any commercial or financial relationships that could be construed as a potential conflict of interest.

## Publisher’s Note

All claims expressed in this article are solely those of the authors and do not necessarily represent those of their affiliated organizations, or those of the publisher, the editors and the reviewers. Any product that may be evaluated in this article, or claim that may be made by its manufacturer, is not guaranteed or endorsed by the publisher.

## Footnotes

1
https://itol.embl.de/
